# Toward metacognition: subject-aware contrastive deep fusion representation learning for EEG analysis

**DOI:** 10.1007/s00422-023-00967-8

**Published:** 2023-07-04

**Authors:** Michael Briden, Narges Norouzi

**Affiliations:** 1 Baskin Engineering, UC Santa Cruz, 1156 High Street, Santa Cruz, CA 95064 USA; 2 Electrical Engineering and Computer Sciences Department, UC Berkeley, Soda Hall, Berkeley, CA 94709 USA

**Keywords:** Electroencephalogram, Deep learning, Fusion, Contrastive representation learning

## Abstract

We propose a subject-aware contrastive learning deep fusion neural network framework for effectively classifying subjects’ confidence levels in the perception of visual stimuli. The framework, called *WaveFusion*, is composed of lightweight convolutional neural networks for per-lead time–frequency analysis and an attention network for integrating the lightweight modalities for final prediction. To facilitate the training of WaveFusion, we incorporate a subject-aware contrastive learning approach by taking advantage of the heterogeneity within a multi-subject electroencephalogram dataset to boost representation learning and classification accuracy. The WaveFusion framework demonstrates high accuracy in classifying confidence levels by achieving a classification accuracy of 95.7% while also identifying influential brain regions.

## Introduction

Electroencephalography (EEG) is a noninvasive and cost-effective method for monitoring brain activity by collecting voltage differences between electrodes placed on a subject’s scalp. EEG signals offer a high temporal resolution, which is well suited for monitoring fast brain processes such as perceptual decision-making. Understanding the metacognition process using EEG signals is an important problem in neuroscience. Confidence has been shown to boost serial dependence in orientation estimation, while subjective confidence is correlated with objective accuracy (Samaha et al. 2019; Fleming et al. 2010). Moreover, confidence is a predictor of performance and of learning motor skills (Rosenqvist and Skans 2015; Stevens et al. 2012). Therefore, by incorporating a subject’s confidence into EEG-based brain–computer interface (BCI) models, a richer understanding of a subject’s perceptive state can be achieved while also understanding the quantity and quality of the evidence supporting the subject’s actions (Yeung and Summerfield 2012).

Stimulation of the visual cortex has been linked to alpha-band power oscillations in humans (Brandt and Jansen 1991; Rajagovindan and Ding 2011). Samaha et al. show that pre-stimulus alpha-band is negatively correlated with subjective confidence ratings in classification tasks (Samaha et al. 2017). Likewise, centro-parietal EEG activity has been linked with perceived decision evidence and confidence during decision-making tasks (Herding et al. 2019). Selimbeyoglu et al. state that an individual’s subjective confidence levels during decision-making tasks can be classified through time and time–frequency analysis of EEG signals and that EEG can be useful in the detection of subjective confidence in single trials (Selimbeyoglu et al. 2012). Based on these results, we work toward an explainable deep learning (DL) model to classify confidence levels in subjects while they identify visual stimuli.

In recent years, the number of DL approaches for classifying EEG data and understanding cognitive tasks has grown, and DL models are regularly used with BCIs to classify neurological signals (Craik et al. 2019). Deng et al. create a BCI containing a convolutional neural network (CNN) for classifying motor-imagery (MI) EEG signals (Deng et al. 2021). Similarly, Dai et al. use a combination of CNNs and auto-encoders (AEs) to classify MI EEG data (Dai et al. 2019). Willet et al. create a BCI for generating text from neural representations of handwriting using recurrent neural networks (RNN) (Willett et al. 2021).

In addition to BCI applications, DL models are used to classify mental states. Al-Ezzi et al. use a CNN long short-term memory (CNN-LSTM) model to classify a subject’s social anxiety from EEG signals (Al-Ezzi et al. 2021). Bălan et al. combine DL and EEG input in a virtual reality system for detecting fear levels and providing treatment for acrophobia (Bălan et al. 2020). DL models are also used to detect ictal EEG signals (Yuan et al. 2018; Cho and Jang 2020; Tsiouris et al. 2018).

While DL has been effectively employed in many neuroscience applications, challenges remain with the quality and availability of the data (Banville et al. 2021; Younes 2017; Huang and Ma 2021). Recently, contrastive learning has shown to be an effective self-supervised learning (SSL) technique to address the issues of the limitation of the data availability, noisy labels, and noisy data (He et al. 2020; Zbontar et al. 2021; Grill et al. 2020). Contrastive learning is often used as a pretext learning task in which DL models are trained to group learned representations of inputs based on features within the input. Such contrastive learning approaches have been used to learn representations from EEG data. Banville et al. use contrastive learning to extract similar features in windowed EEG data (Banville et al. 2019). Citing subject-invariant representations of emotion, Shen et al. explore the use of contrastive learning to train a DL model to extract cross-subject emotion representations (Shen et al. 2022). Han et al. also use self-supervised contrastive learning to label EEG data for motor imagery classification (Han et al. 2021). Contrastive learning has also increased DL model generalizability with a limited sample size (Jiang et al. 2021). Conversely, Kostas et al. use contrastive learning to aid DL models in learning massive amounts of unlabeled EEG data (Kostas et al. 2021).

**Fig. 1:**
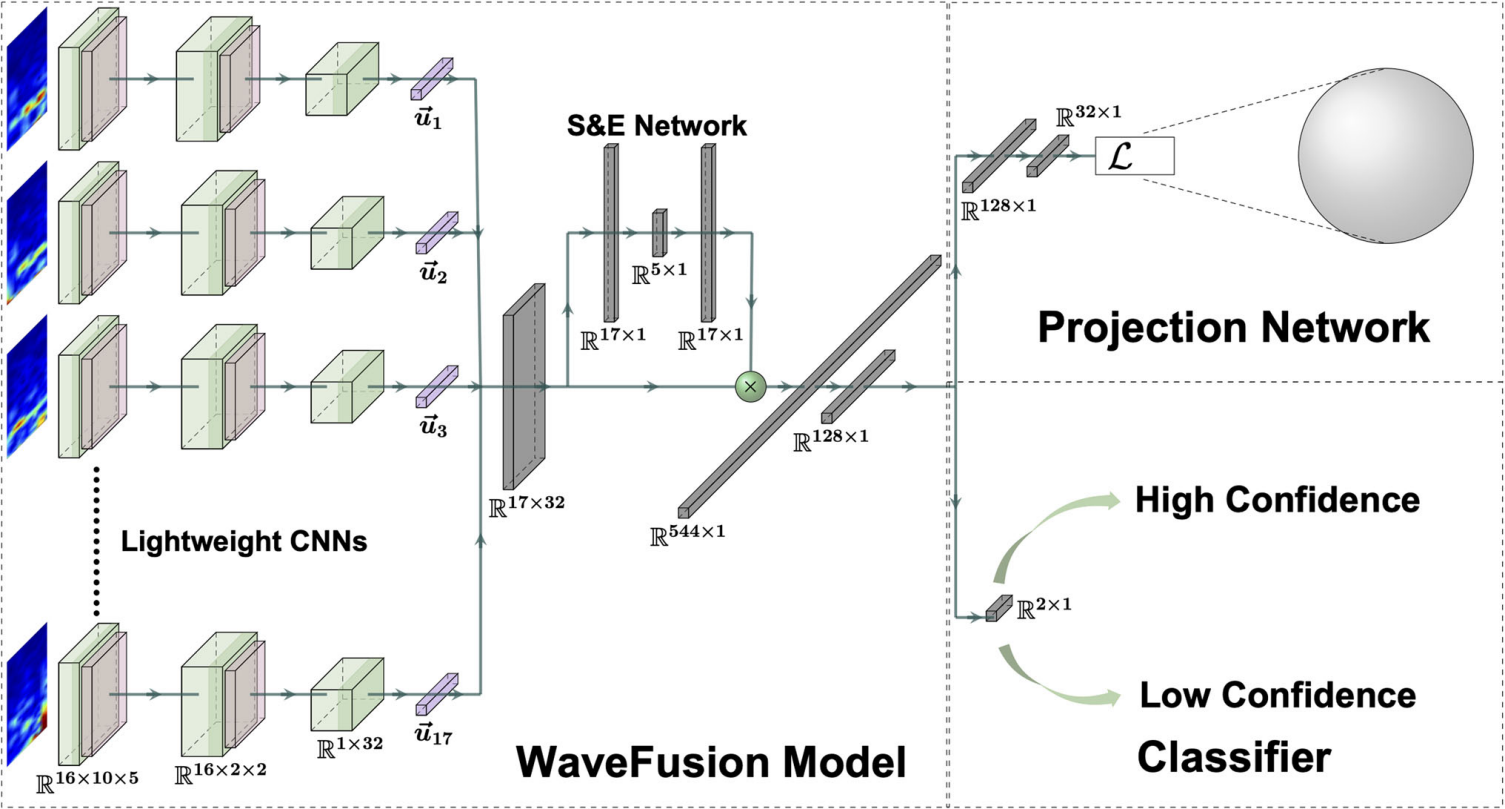
The WaveFusion architecture contains 17 lightweight CNNs that learn the time–frequency features of a specific EEG lead. During the subject-aware contrastive learning (SAC) training phase, $128\times 1$ feature maps are forwarded to the projection network and embedded into the unit sphere via SAC loss. During classification, the learned embeddings are sent to a classification layer

Based on the principle that, for supervised learning tasks, increasing the amount of information in input data boosts a machine learning (ML) model’s generalizability, multimodal fusion models have gained popularity as effective means for dealing with limited or noisy data. Fusion models typically consist of multiple ML models that learn data sampled from different modes to predict the same outcome. Multimodal models are commonly studied for detecting neurological disorders. Alghowinem et al. combine linguistic queues (audio data), head pose (image data), and eye gaze to monitor a subject’s depression level (Alghowinem et al. 2016). Wristbands that capture multimodal data such as electrodermal activity and accelerometer data have been used to train fusion models to detect Parkinson’s disease and detect seizures (Regalia et al. 2019; Onorati et al. 2017). EEG-specific fusion models have also been created to analyze neurological events. Briden et al. create a multimodal fusion model that combines the outputs of 61 CNNs, each trained on data from a specific EEG lead, to infer anxiety level (Briden and Norouzi 2021). Cai et al. train a classifier on EEG data generated by subjects listening to positive or negative audio stimuli to detect depression level (Cai et al. 2020).

In this work, we combine multimodal fusion and contrastive learning in order to create an explainable DL model to infer a subject’s confidence in the perception of visual stimuli. We train a multimodal *WaveFusion Squeeze and Excite (WaveFusion)* (shown in Fig. 1) network through a contrastive learning feature extraction approach (Briden and Norouzi 2021). We first learn relevant short-time Fourier transform (STFT) features by mapping STFT data to a 32-dimensional hyper-sphere using a *WaveFusion with a Projection Network (WFP)*. WFP’s weights are then transferred to a *WaveFusion Classifier (WFC)* where STFT data are classified as either low or high confidence.

## Dataset

The EEG data in this experiment contain readings from 25 subjects undergoing a perceptual decision-making task (Samaha and Cohen 2022). The data are collected from 63 leads and one ground arranged in the 10-20 localization system. Subjects viewed a $300\ ms$ dot-motion stimulus (left or right) with six interleaved levels of motion coherence ranging between 0.01, 0.045, 0.08, 0.12, 0.25, and 0.4. The subjects indicated their perceived direction of motion and their confidence in their response on a scale from 1 to 4 (1 indicates the lowest and 4 is the highest confidence level). The fraction of dot during the initial cleaning, trials with excessive noise were removed, and recordings were down-sampled from 4000 to 500 Hz. We selected recordings from 10 subjects with at least 60 high- and 60 low-confidence recordings. Half of the trials from each subject were used for training, and the other half was reserved for validation and testing.

We trained the WaveFusion architecture to recognize high or low confidence levels in this analysis. We selected ten subjects who had over a hundred high and low recordings. We then split each subject’s recordings into high and low samples into training and test sets. For each subject, we created 1000 averaged EEG recordings by averaging over 25 randomly selected EEG samples from the subject’s high training set samples (500 generated high-confidence samples) and the subject’s low-confidence training set (500 generated low-confidence samples). Likewise, the test set is comprised of 250 averaged samples for each patient and confidence level. We then truncated each sample to stimulus onset to 1 second afterward, down-sampled to 100 Hz, and applied STFT to each sample with a window size of 80 Hz and overlap of $75\%$ yielding 63 spectrograms (one for each EEG lead) of size $39 \times 11$.

To limit the size and scope of the WaveFusion framework, we concatenated the posterior spectrograms to create $17 \times 39\times 11$ tensors which are forwarded to the models. Samaha et al. showed that a cluster of posterior electrodes displays high levels of alpha-band power between $-500\ ms$ to stimulus onset (when averaged across all trials), which biases decision confidence (Samaha et al. 2017). Moreover, parietal error positivity (PE) has been linked to error monitoring and influencing decision confidence, while the parietal cortex has been shown to play a role in decision confidence in rhesus monkeys (Falkenstein et al. 1990; Kiani et al. 2009; Boldt and Yeung 2015).

**Table 1:** Architectures and parameters for the LWCNN and SEN modules

Operation	Kernel	Strides	Padding	Count	BN?	Dropout	Nonlinearity
LWCNN: $1\times 39\times 11$ input							
2D Convolution	$5\times 4$	$2\times 1$	$2\times 1$	16	$\times$	0.1	ReLU
2D max-pooling	$2\times 2$			16	$\times$		
2D convolution	$4\times 2$	$2\times 1$		16	$\checkmark$	0.1	ReLU
2D max-pooling	$2\times 2$			16	$\times$		
2D convolution	$2\times 2$	$1\times 1$		32	$\checkmark$	0.1	
SEN: $17\times 32$ input							
Linear	N/A	N/A	N/A	17	$\times$		ReLU
Linear	N/A	N/A	N/A	5	$\times$		ReLU
Linear	N/A	N/A	N/A	17	$\times$		Sigmoid

## Models and algorithms

To learn latent space embeddings for STFT information, we employ a WaveFusion framework which contains 17 individual CNNs trained on the STFT data specific to a single EEG lead (Fig. 1). The feature maps from the CNNs are combined using a lightweight Squeeze and Excite Attention Network (SEN) before being embedded into the unit hyper-sphere or classified. This section describes the WaveFusion framework, including WaveFusion Projection (WFP) network and WaveFusion Classifier (WFC), and the subject-aware contrastive learning (SAC) framework.

### WaveFusion projection network

*Lightweight Convolutional Neural Networks:* The WaveFusion models take in spectrogram tensors where each spectrogram is forwarded to 1 of the 17 lightweight 2D-CNNs (LWCNNs). Each LWCNN learns features of a spectrogram generated by one EEG lead using three convolution layers. The first two layers are followed by ReLU activation, $2 \times 2$ max-pooling, a dropout layer with a fixed drop rate of $10\%$, and batch normalization (see Table 1). The last convolution layer is followed by a fixed $10\%$ dropout layer with batch normalization and outputs a feature map of size $1 \times 32$.

As opposed to full-sized contemporary CNNs such as ResNet, DenseNet, and Inception-V4, which can have up to 50 convolutional layers, the LWCNNs contain only three layers and are tasked with recognizing features specific to an EEG lead rather than being charged with identifying a host of natural images (Targ et al. 2016; Hasan et al. 2021; Szegedy et al. 2017).

*Squeeze and Excite Network:* A lightweight attention module is used to weigh the feature maps generated by the 17 LWCNNs. Attention modules are often used in image segmentation models and allow for CNN architectures to focus on essential details in an image by up- and down-weighting CNN activations (Vaswani et al. 2017). Hu et al. propose a lightweight attention network called “squeeze and Excitation Network” (SEN), which can be used in between DL model layers in order to up-weight important CNN channels before they are sent to the next CNN layer (Hu et al. 2018).

The 17 LWCNN outputs are combined to form the tensor of feature maps $\varvec{U}=\left[ \varvec{u}_1,\varvec{u}_2,...\varvec{u}_{17} \right] \in \mathbb {R}^{17\times 32}$, which SEN uses to generate attention weight, $\pi _i$, for each map. Table 1 outlines the SEN architecture. SEN consists of a global pooling layer that reduces $\varvec{U}$ to a $17\times 1$ tensor of averages, which are sent to an encoder–decoder model that contains two dense layers. The dense encoder layer condenses the $17\times 1$ input to $5\times 1$, followed by ReLU activation. The dense decoder layer expands the output back to $17\times 1$ where sigmoid activation with a temperature parameter computes attention weights, $\pi _i$. WaveFusion architectures tend to over-fit when the SEN over-emphasizes a small number of channels while down-weighting others. To address this issue, the weights are flattened by using temperature $\tau$ within the sigmoid activation function:

1 $$\begin{aligned} \pi _i=\frac{e^{z_i/\tau }}{e^{z_i/\tau }+1} \end{aligned}$$

where $z_i$ is the summed and weighted input to the last fully connected layer (Chen et al. 2020). This flattening drives probability scores toward 0.5 and allows for even optimization across the LWCNN models. The SEN, along with the lightweight CNNs, can lead to the localization of neural activities in the human brain.

*Subject-aware contrastive learning framework* typical contrastive learning methodologies group like representations by class or similarity Khosla et al. (2020). However, these techniques do not account for multiple conditions generated from a single subject, such as high- and low-confidence recordings generated from a single subject. As such, we develop a subject-aware contrastive learning framework that contrasts data from a given condition and subject with the rest of the data. We outline our process below.

First, we make use of three components commonly used to facilitate contrastive learning: *Data Augmentation, Aug()*: For each $\vec {x}$ in a batch, two augmentations, $\tilde{x}$ (called “views” of $\vec {x}$), are generated by augmenting $\vec {x}$ with Gaussian pink noise, input dropout, and random Gaussian noise.

**Table 2:** Summary of the impact of subject-aware batch construction and SAC loss function on the confidence level classification task. Column 1 shows the batch construction scheme (percentages of intra-subject negatives, inter-subject negatives, and positives). Columns 2 to 5 summarize the F1 score and accuracy for low- and high-confidence conditions. Column 6 shows the overall classification accuracy

*Q*(*i*) / $N(i)_{r}$ / $N(i)_{a}$ (%)	Low		High		Overall
	**F1**	Accuracy	F1	Accuracy	Accuracy
SupCon Loss	92.3	95.1	91.8	89.0	92.0
12.5/37.5/50	91.3	89.4	91.7	91.6	91.5
25/25/50	91.3	95.4	90.6	86.6	91.0
37.5/12.5/50	92.7	95.3	92.3	89.7	92.5
45/5/50	92.1	97.4	91.1	85.8	91.6
5/45/50	93.1	97.1	92.5	88.5	92.8
50/0/50	**95**.**8**	**98**.**7**	**95**.**6**	**92**.**7**	**95**.**7**

The best performing batch construction is highlighted in bold

*Encoder Network, Enc()*: the encoder model is a combination of LWCNNs, SEN, a flattening layer, and a dense layer to map inputs $\tilde{x}$ to a vector $\vec {r}=Enc(\tilde{x})\in \mathbb {R}^{128}$.

*Projection network, Proj()*: Specific to WFP, representations are sent to the projection network that maps $\vec {r}$ to an embedding vector $\vec {z}=Proj(\vec {r})\in \mathbb {R}^{32}$. The embedding $\vec {z}$ is then normalized to a unit hyper-sphere in $D_{32}$, which allows for measuring cosine similarity between projections.

We define a multi-label batch of *N* randomly sampled pairs as $\mathcal {B}=\{\vec {x}_k,\vec {y}_{1,k},\vec {y}_{2,k}\}_{k=1,..., N}$ where $\vec {y}_{1,k}\in \{0,1\}$ denotes the confidence label and $\vec {y} _{2,k}\in \{1,10\}$ is a unique subject ID label (Khosla et al. 2020). Augmentation is applied to each STFT tensor in $\mathcal {B}$ to create the new batch of views $\mathcal {B}_s=\{\tilde{x}_k,\tilde{y}_{1,k},\tilde{y}_{2,k}\}_{k=1,..., 2N}$ where $\tilde{y}_{1,2k-1}=\tilde{y}_{1,2k}=\vec {y}_{1,k}$ and $\tilde{y}_{2,2k-1}=\tilde{y}_{2,2k}=\vec {y}_{2,k}$. Letting $i\in I_s=\{1,..., 2N\}$ be the index of an arbitrary sample in $\mathcal {B}_s$, we define the SAC loss as

2 $$\begin{aligned} \mathcal {L}=-\sum _{i\in I_s}log\left( \frac{1}{\Vert Q(i)\Vert }\sum _{q\in Q(i)} \frac{exp\left( \vec {{\textbf {z}}_i}\cdot \vec {{\textbf {z}}_q}/\tau \right) }{\sum _{s\in S(i)}exp\left( \vec {{\textbf {z}}_i} \cdot \vec {{\textbf {z}}_s}/\tau \right) } \right) \nonumber \\ \end{aligned}$$

where $\vec {{\textbf {z}}_i}$ serves as the anchor with $\tau \in \mathbb {R}^+$ as a temperature parameter. The set of positives, $Q(i)=\{q \in I_s-\{i\}:\tilde{y}_{1,q} = \tilde{y} _{1,i} \text {\ and\ } \tilde{y}_{2,q} = \tilde{y} _{2,i} \}$, contains all samples generated from the same subject and of the same class as the anchor. The set $S(i)= N(i)_{r} \cup N(i)_{a}$ is the set of negatives with $N(i)_{r}=\{s \in I_s-\{i\}:\tilde{y}_{1,s} \ne \tilde{y} _{1,i} \text {\ and\ } \tilde{y}_{2,s} \ne \tilde{y} _{2,i} \}$ containing the inter-subject samples with the opposite class label compared to the anchor’s and $N(i)_{a}=\{s \in I_s-\{i\}:\tilde{y}_{1,s} \ne \tilde{y} _{1,i} \text {\ and\ } \tilde{y}_{2,s} = \tilde{y} _{2,i} \}$ containing intra-subject samples with the opposite class label compared to the anchor’s.

One training epoch is completed when each sample in the training set serves as the anchor. Thus, we aim to strategically alter the composition of $\mathcal {B}$ and subsequently *Q*(*i*), $N(i)_{r}$, and $N(i)_{a}$ in order to study the effects of SAC on inferring subject’s confidence levels with the WaveFusion architecture.

### WaveFusion classification network

After WFP has been trained, the WaveFusion weights are transferred to a WFC model for the classification task. A dense classification layer with two nodes follows the WaveFusion architecture to generate the logits required for softmax classification. Spectrograms are fed to WFC in a standard manner without augmentation, and WFC’s weights are fine-tuned to achieve optimal classification.

**Fig. 2:**
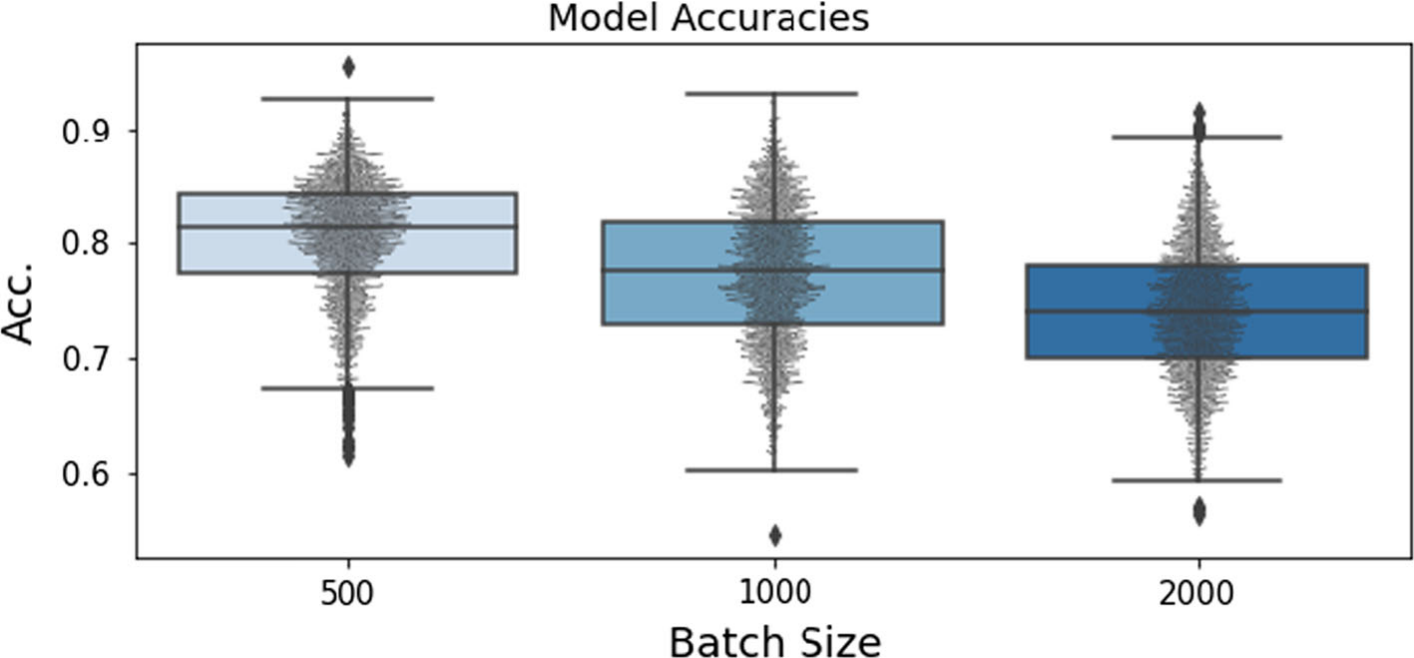
Box and swarm plots of the model classification accuracy for given batch sizes. Each dot represents the best accuracy achieved by a model during grid search

## Experiments and results

We conduct multiple experiments to study the effects of batch formulation on the WaveFusion framework, and localization effects of the WFC model, and study the impact of varying model configurations to gauge the impact of SAC pre-training and SEN mechanism.

### Batch construction

To observe the effect of the SAC training scheme and the associated batch construction, we experimented with the percentage of positive (same class from the same subject) and negative samples (intra-subject and inter-subject negatives—other class from the same subject and other subjects, respectively) in each batch. We also experimented with the impact of the batch size and other model parameters to find the best embedding for the confidence level classification task. The accuracy and F1 score of the confidence level classification task for different levels of intra- and inter-subject negative batch construction are shown in Table 2 (Hicks et al. 2022). The subject-aware WaveFusion model has $98.7\%$ and $92.7\%$ classification accuracy for low- and high-confidence classes. The model also outperforms the same architecture trained using a standard supervised contrastive loss function (row 1 of Table 2).

**Fig. 3:**
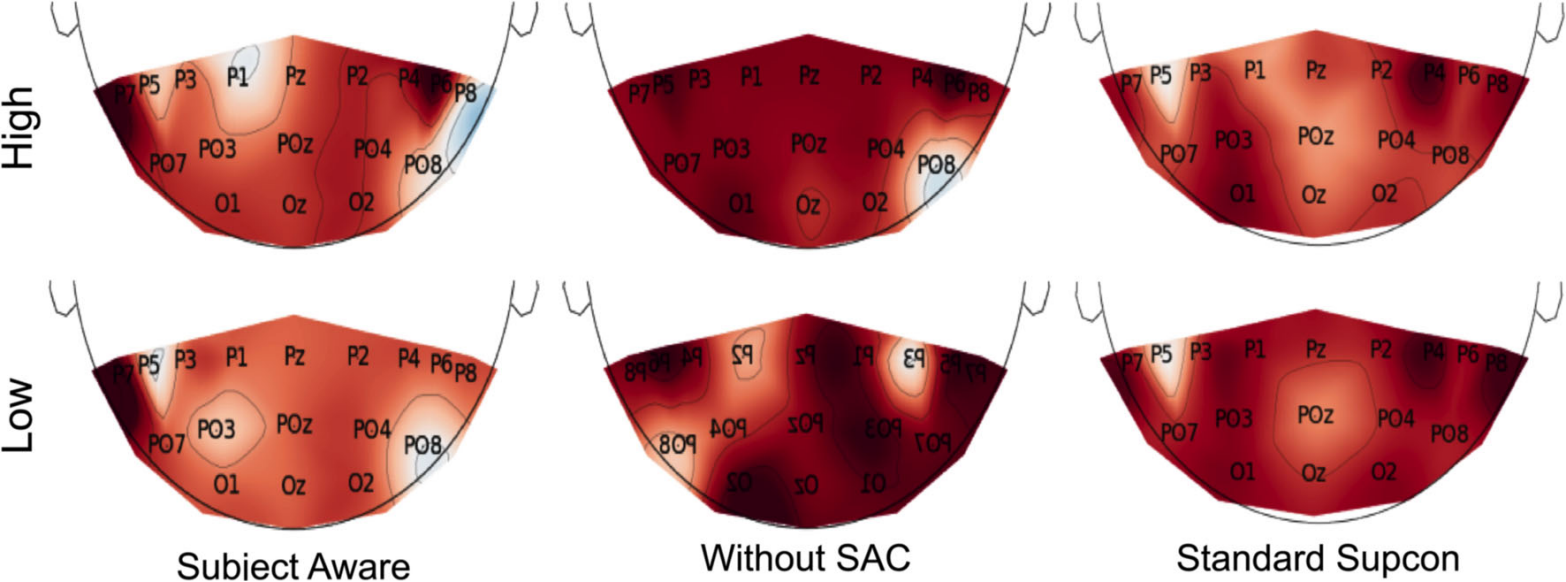
Interpolation of most influential modalities onto a 2D topology maps. Column 1 shows the interpolations generated by the SAC pre-trained WFC, column 2 shows the interpolations generated by a WFC without pre-training, and column 3 shows the interpolations generated by a WFC pre-trained with standard supervised contrastive learning

When experimenting with different batch sizes, we considered all combinations of hyper-parameters, such as SAC loss temperature and weight decay. Each WFP is trained for 25 epochs with a fixed learning rate of 0.05. We then transfer the weights to WFC and perform a grid search over the weight decay hyper-parameter with Adam optimizer with a learning rate of $1\times 10^{-4}$ and train each model for 150 epochs. A WFP model using a batch size of 500 with 250 positives, 250 intra-subject negatives, no inter-subject negatives, loss temperature of 0.25, and weight decay of $7\times 10^{-3}$ led to the best overall WFC classification accuracy of $95.7\%$ (see Table 2).

Additionally, to study the impact of the batch size, we trained the model using batches of sizes 500, 1000, and 2000. As shown in Fig. 2, the model performs best with the batch size of 500.

### Localizing neurological activities

After the WFC models are trained, we use the SEN properties to identify influential brain activity regions. We then compare the class activation maps generated by four LWCNNs. A sample from the low-confidence and high-confidence classes are used for illustration (Zhou et al. 2016). As described below, the WFC models pre-trained with SAC show a higher capability for localizing brain activity. Our proposed approach can localize brain activity at inference time without any additional post-processing.

To understand the impact of the contrastive learning training scheme in localizing neurological activities, we used the 17 attention weights, $\pi _i$, generated during the inference phase and interpolated them onto brain and scalp models. The attention weights display desirable properties for comparing activity across regions. Since the weights are learned during model training, the influence of each LWCNN has adjusted automatically. Moreover, the SEN attention weights are proportional to the amount of activation in each channel, but are not prone to over-optimizing channel-specific details. Likewise, the SEN attention weights are limited to between 0 and 1, allowing for a fair comparison across modalities.

Column 1 of Fig. 3 illustrates the interpolation of attention weights corresponding to a high- and a low-confidence recording generated from the subject-aware contrastive learning WFC. In the low-confidence example, we see considerable influence from P7 lead (indicated by dark red areas). In the high-confidence example, influence is generated largely by P7 and P6 leads.

**Table 3:** Component analysis on SEN and SAC pre-training. Classification accuracy and F1 score are given for each model configuration

Configuration	Low Confidence		High Confidence		Overall
	F1	Accuracy	F1	Accuracy	Accuracy
SupCon Loss	92.3	95.1	91.8	89.0	92.0
Without SAC	86.8	92.7	84.8	79.0	85.8
Without SEN	91.8	94.2	91.4	89.0	91.6
No SAC, No SEN	90.8	91.7	90.6	89.8	90.7
SAC	**95**.**8**	**98**.**7**	**95**.**6**	**92**.**7**	**95**.**7**

The best performance for each metric is highlighted in bold

**Fig. 4:**
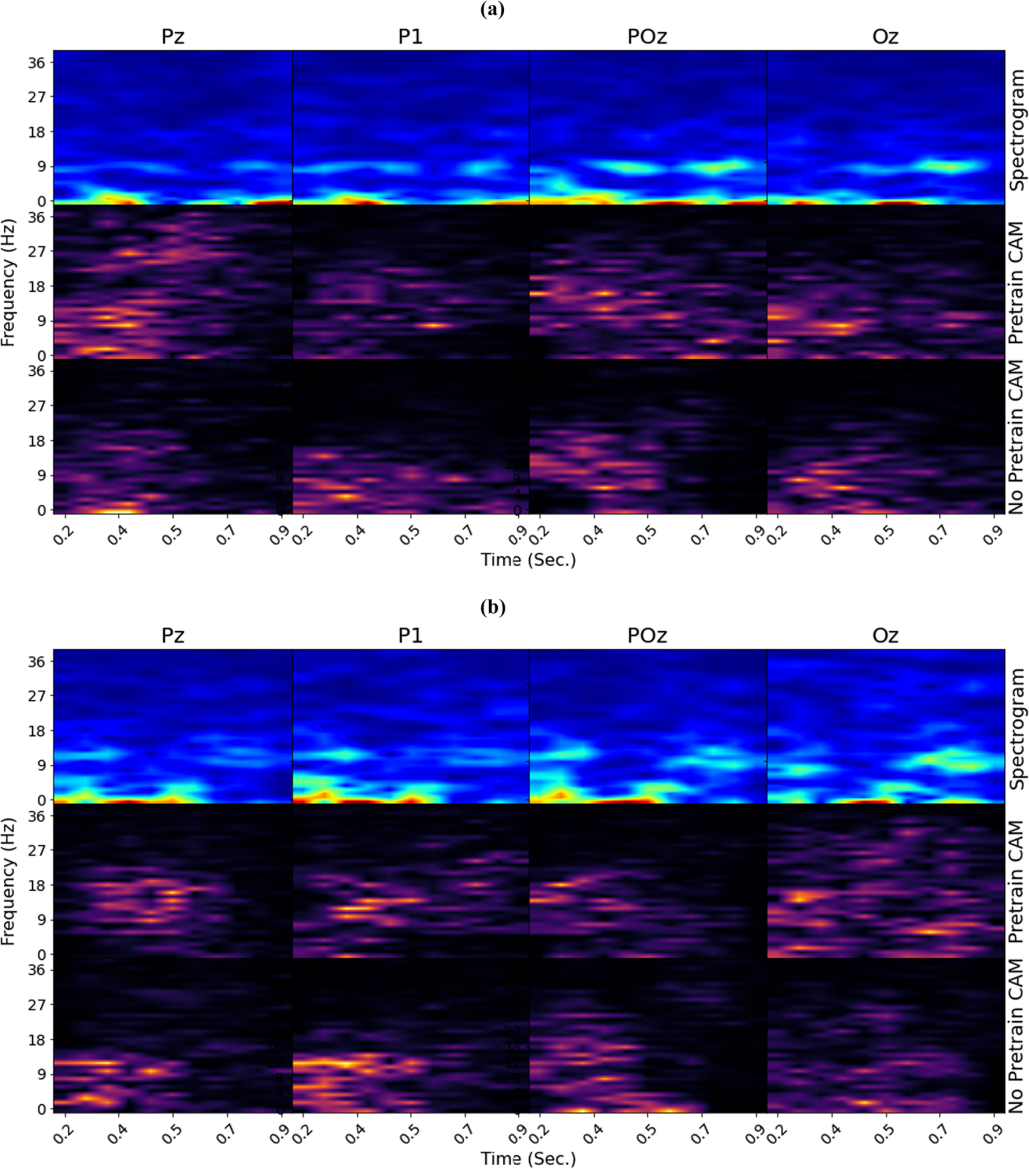
**a** Class activation maps generated using a low-confidence example, by the WFC pre-trained with the subject-aware contrastive loss (middle row) and without pre-training (bottom row) for a low-confidence example. **b** Class activation maps generated using a high-confidence example

### Network evaluation

To evaluate the impact of different components on the overall model performance, we create different configurations of the WaveFusion model by removing components to observe the effects of SAC pre-training and SEN on the WaveFusion framework. Table 3 shows the classification results for the different configurations used. When removing SAC pre-training, WFC only achieves a classification accuracy of $85.8\%$ percent. Likewise, when the SEN is removed from the WaveFusion framework, WFC achieves a classification accuracy of $91.6\%$, which suggests that SAC pre-training boosts the altered model’s performance. Lastly, by removing SEN and SAC pre-training from the framework, the WFC achieves a classification accuracy of $90.7\%$.

Figure 3 column 2 visualizes the interpolated attention weights from a WFC model without pre-trained attention weights. In contrast, column 3 visualizes the weights from a WFC pre-trained with standard supervised contrastive (SupCon) learning. The interpolation for the non-pre-trained WFC indicates that the model considers a high level of attention from multiple modalities compared to the SAC pre-trained model, which suggests that the model struggles to localize activity. The interpolated weights from the WFC pre-trained with standard SupCon suggest a moderate level of localization. Yet, the localization is not as clear as the SAC pre-trained model. Figure 4 provides a visual comparison between CAMS from WFC models with SAC pre-trained weights and non-pre-trained WFC for the low-confidence (a) and high-confidence (b) examples. The middle rows of Figs. 4.a show the CAMs generated by LWCNNs from a WFC with pre-trained SAC weights while the bottom rows show the CAMs from a non-pre-trained WFC for the low-confidence input. 4.b shows the CAMS generated using a high-confidence example. The CAMs generated from the non-pre-trained LWCNNs suggest that the model focuses on a time range of 0.0 to 0.5 seconds and a frequency range of 0 to 18 Hz on the low-confidence example. The CAMs generated from the pre-trained LWCNNs appear to learn a richer set of features with activation covering a larger time and frequency range of $0-36$ Hz, which suggests that the LWCNNs detect Beta oscillations correlated with perceptual judgment in addition to the pre-stimulus Alpha activity found by Samaha (Haegens et al. 2017; Samaha et al. 2017).

**Table 4:** Accuracies and F1 Score for the motion coherence classification task

Low coherence (0.01)		High Coherence (0.4)		Overall
F1	Accuracy	F1	Accuracy	Accuracy
84.5	78.1	86.7	93.3	85.7

### Detecting type 1 behavioral response

In addition to confidence level classification, we ran experiments to determine whether WaveFusion could detect a Type 1 behavior response: whether the dots moved left or right. Likewise, we set up a binary classification task to determine whether WaveFusion could distinguish between motion coherence of 0.01 and 0.4 from the EEG signals. The data were constructed using the methodology outlined in Sect. 2. While the direction detection experiment was inconclusive, the WaveFusion architecture could detect motion coherence with an appreciable degree of accuracy of $85.7\%$ when using a batch size of 500, and no inter-subject negatives. Table 4 shows the classification results and F1-score.

## Conclusion

The proposed WaveFusion framework, along with the SAC training scheme, is an important step toward 1) multimodal EEG analysis through an attention-based fusion technique and 2) using a SAC training scheme for an EEG classification task. The framework illustrates the impact of building an EEG contrastive learning representation learning scheme in a subject-aware manner to boost feature learning and classification accuracy. We show that a batch comprising $50\%$ positives, $50\%$ intra-subject negatives, and no inter-subject negatives yields the best results during the classification task. Moreover, we show that SEN displays important properties for localizing neurological events and that SAC pre-training reduces WaveFusion’s sensitivity to background neurological events. We then demonstrate that WaveFusion can detect motion coherence to an appreciable degree of accuracy which suggests that the architecture may be tailored for detecting Type 1 Behavioral responses. Lastly, we show that the WaveFusion architecture effectively learns EEG representations in an interpretable manner, using lead-specific lightweight CNNs and attention to localize neural activities in the human brain.

## References

[CR1] Al-Ezzi A, Yahya N, Kamel N (2021). Severity assessment of social anxiety disorder using deep learning models on brain effective connectivity. IEEE Access.

[CR2] Alghowinem S, Goecke R, Wagner M (2016). Multimodal depression detection: fusion analysis of paralinguistic, head pose and eye gaze behaviors. IEEE Trans Affect Comput.

[CR3] Banville H, Albuquerque I, Hyvärinen A, et al (2019) Self-supervised representation learning from electroencephalography signals. In: 2019 IEEE 29th International Workshop on Machine Learning for Signal Processing (MLSP), pp 1–6, 10.1109/MLSP.2019.8918693

[CR4] Banville H, Chehab O, Hyvärinen A (2021). Uncovering the structure of clinical EEG signals with self-supervised learning. J Neural Eng.

[CR5] Boldt A, Yeung N (2015). Shared neural markers of decision confidence and error detection. J NeuroscI.

[CR6] Brandt ME, Jansen BH (1991). The relationship between prestimulus alpha amplitude and visual evoked potential amplitude. Int J Neurosci.

[CR7] Briden M, Norouzi N (2021) WaveFusion Squeeze-and-Excitation: Towards an Accurate and Explainable Deep Learning Framework in Neuroscience. In: 2021 43rd Annual International Conference of the IEEE Engineering in Medicine & Biology Society (EMBC), pp 1092–1095, 10.1109/EMBC46164.2021.9630605 10.1109/EMBC46164.2021.9630605 34891477

[CR8] Bălan O, Moise G, Moldoveanu A (2020). An investigation of various machine and deep learning techniques applied in automatic fear level detection and Acrophobia virtual therapy. Sensors.

[CR9] Cai H, Qu Z, Li Z (2020). Feature-level fusion approaches based on multimodal eeg data for depression recognition. Inf Fusion.

[CR10] Chen Y, Dai X, Liu M, et al (2020) Dynamic convolution: Attention over convolution kernels. In: Proceedings of the IEEE/CVF Conference on Computer Vision and Pattern Recognition, pp 11,030–11,039

[CR11] Cho KO, Jang HJ (2020). Comparison of different input modalities and network structures for deep learning-based seizure detection. Sci Rep.

[CR12] Craik A, He Y, Contreras-Vidal JL (2019). Deep learning for electroencephalogram (eeg) classification tasks: a review. J Neural Eng.

[CR13] Dai M, Zheng D, Na R (2019). Eeg classification of motor imagery using a novel deep learning framework. Sensors.

[CR14] Deng X, Zhang B, Yu N, et al (2021) Advanced TSGL-EEGNet for Motor Imagery EEG-Based Brain-Computer Interfaces. IEEE Access 9:25,118–25,130. 10.1109/ACCESS.2021.3056088

[CR15] Falkenstein M, Hohnsbein J, Hoormann J, et al (1990) Psychophysiological brain research. Tilburg University Press, Tilburg pp 192–195

[CR16] Fleming SM, Weil RS, Nagy Z (2010). Relating introspective accuracy to individual differences in brain structure. Science.

[CR17] Grill JB, Strub F, Altché F (2020). Bootstrap your own latent-a new approach to self-supervised learning. Adv Neural Inf Process Syst.

[CR18] Haegens S, Vergara J, Rossi-Pool R (2017). Beta oscillations reflect supramodal information during perceptual judgment. Proc Nat Acad Sci.

[CR19] Han J, Gu X, Lo B (2021) Semi-supervised contrastive learning for generalizable motor imagery eeg classification. In: 2021 IEEE 17th International Conference on Wearable and Implantable Body Sensor Networks (BSN), pp 1–4, 10.1109/BSN51625.2021.9507038

[CR20] Hasan N, Bao Y, Shawon A (2021). Densenet convolutional neural networks application for predicting covid-19 using ct image. SN Comput Sci.

[CR21] He K, Fan H, Wu Y, et al (2020) Momentum contrast for unsupervised visual representation learning. In: Proceedings of the IEEE/CVF conference on computer vision and pattern recognition, pp 9729–9738

[CR22] Herding J, Ludwig S, von Lautz A (2019). Centro-parietal eeg potentials index subjective evidence and confidence during perceptual decision making. NeuroImage.

[CR23] Hicks SA, Strümke I, Thambawita V (2022). On evaluation metrics for medical applications of artificial intelligence. Sci Rep.

[CR24] Hu J, Shen L, Sun G (2018) Squeeze-and-excitation networks. In: Proceedings of the IEEE conference on computer vision and pattern recognition, pp 7132–7141

[CR25] Huang G, Ma F (2021) Concad: Contrastive learning-based cross attention for sleep apnea detection. In: Joint European Conference on Machine Learning and Knowledge Discovery in Databases, Springer

[CR26] Jiang X, Zhao J, Du B, et al (2021) Self-supervised contrastive learning for eeg-based sleep staging. In: 2021 International Joint Conference on Neural Networks (IJCNN), IEEE, pp 1–8, 10.1109/IJCNN52387.2021.9533305

[CR27] Khosla P, Teterwak P, Wang C, et al (2020) Supervised Contrastive Learning. In: Advances in NeuralInformationProcessingSystems, vol 33. Curran Associates, Inc., pp 18,661–18,673

[CR28] Kiani R, Shadlen MN (2009). Representation of confidence associated with a decision by neurons in the parietal cortex. Science.

[CR29] Kostas D, Aroca-Ouellette S, Rudzicz F (2021) Bendr: using transformers and a contrastive self-supervised learning task to learn from massive amounts of eeg data. Front Human Neurosci 253 10.3389/fnhum.2021.653659 PMC8261053 34248521

[CR30] Onorati F, Regalia G, Caborni C (2017). Multicenter clinical assessment of improved wearable multimodal convulsive seizure detectors. Epilepsia.

[CR31] Rajagovindan R, Ding M (2011). From prestimulus alpha oscillation to visual-evoked response: an inverted-u function and its attentional modulation. J Cognit Neurosci.

[CR32] Regalia G, Onorati F, Lai M (2019). Multimodal wrist-worn devices for seizure detection and advancing research: focus on the empatica wristbands. Epilepsy Res.

[CR33] Rosenqvist O, Skans ON (2015). Confidence enhanced performance?—The causal effects of success on future performance in professional golf tournaments. J Econ Behav Org.

[CR34] Samaha J, Cohen MX (2022). Power spectrum slope confounds estimation of instantaneous oscillatory frequency. NeuroImage.

[CR35] Samaha J, Iemi L, Postle BR (2017). Prestimulus alpha-band power biases visual discrimination confidence, but not accuracy. Conscious Cognit.

[CR36] Samaha J, Switzky M, Postle BR (2019). Confidence boosts serial dependence in orientation estimation. J Vis.

[CR37] Selimbeyoglu A, Keskin-Ergen Y, Demiralp T (2012). What if you are not sure? electroencephalographic correlates of subjective confidence level about a decision. Clin Neurophysiol.

[CR38] Shen X, Liu X, Hu X, et al (2022) Contrastive learning of subject-invariant eeg representations for cross-subject emotion recognition. IEEE Transactions on Affective Computing p 1. 10.1109/TAFFC.2022.3164516

[CR39] Stevens D, Anderson DI, O’Dwyer NJ (2012). Does self-efficacy mediate transfer effects in the learning of easy and difficult motor skills?. Conscious Cogn.

[CR40] Szegedy C, Ioffe S, Vanhoucke V, et al (2017) Inception-v4, inception-resnet and the impact of residual connections on learning. In: Proceedings of the AAAI conference on artificial intelligence

[CR41] Targ S, Almeida D, Lyman K (2016) Resnet in resnet: Generalizing residual architectures. arXiv preprint arXiv:1603.08029

[CR42] Tsiouris KM, Pezoulas VC, Zervakis M (2018). A long short-term memory deep learning network for the prediction of epileptic seizures using eeg signals. Comput Biol Med.

[CR43] Vaswani A, Shazeer N, Parmar N, et al (2017) Attention is all you need. arXiv preprint arXiv:1706.03762

[CR44] Willett FR, Avansino DT, Hochberg LR (2021). High-performance brain-to-text communication via handwriting. Nature.

[CR45] Yeung N, Summerfield C (2012). Metacognition in human decision-making: confidence and error monitoring. Philosophical Transa Royal Soc B: Biol Sci.

[CR46] Younes M (2017). The case for using digital eeg analysis in clinical sleep medicine. Sleep Sci Pract.

[CR47] Yuan Y, Xun G, Jia K (2018). A multi-view deep learning framework for eeg seizure detection. IEEE J Biomed Health Inf.

[CR48] Zbontar J, Jing L, Misra I, et al (2021) Barlow twins: Self-supervised learning via redundancy reduction. In: International Conference on Machine Learning, PMLR, pp 12,310–12,320

[CR49] Zhou B, Khosla A, Lapedriza A, et al (2016) Learning deep features for discriminative localization. In: Proceedings of the IEEE/CVF Conference on Computer Vision and Pattern Recognition, pp 2921–2929

